# Effect of MAX Phase Ti_3_ALC_2_ on the Ultrafiltration Membrane Properties and Performance

**DOI:** 10.3390/membranes13050456

**Published:** 2023-04-24

**Authors:** Tamara Wahid Abood, Kadhum M. Shabeeb, Aseel B. Alzubaydi, Hasan Sh. Majdi, Raed A. Al-Juboori, Qusay F. Alsalhy

**Affiliations:** 1 Department of Materials Engineering, University of Technology-Iraq, Alsinaa Street 52, Baghdad 10066, Iraq; 2 Department of Chemical Engineering and Petroleum Industries, AlMustaqbal University College, Babylon 51001, Iraq; 3 NYUAD Water Research Centre, Abu Dhabi Campus, New York University, Abu Dhabi P.O. Box 129188, United Arab Emirates; 4 Membrane Technology Research Unit, Department of Chemical Engineering, University of Technology-Iraq, Alsinaa Street 52, Baghdad 10066, Iraq

**Keywords:** hydrophilicity, MAX phase Ti_3_ALC_2_ (Ti3C2Tx), ultrafiltration, PVDF, membrane, antifouling

## Abstract

Membrane fouling remains a major obstacle to ultrafiltration. Due to their effectiveness and minimal energy demand, membranes have been extensively employed in water treatment. To improve the antifouling property of the PVDF membrane, a composite ultrafiltration membrane was created employing the in-situ embedment approach throughout the phase inversion process and utilizing a new 2D material, MAX phase Ti_3_ALC_2_. The membranes were described using FTIR (Fourier transform infrared spectroscopy), EDS (energy dispersive spectroscopy), CA (water contact angle), and porosity measurements. Additionally, atomic force microscopy (AFM), field emission scanning electron microscopy (FESEM), and energy dispersive spectroscopy (EDS) were employed. Standard flux and rejection tests were applied to study the produced membranes’ performance. Adding Ti_3_ALC_2_ reduced composite membranes’ surface roughness and hydrophobicity compared to the pristine membrane. Porosity and membrane pore size increased with the addition up to 0.3% *w*/*v*, which decreased as the additive percentage increased. The mixed matric membrane with 0.7% *w*/*v* of Ti_3_ALC_2_ (M7) had the lowest CA. The alteration in the membranes’ properties reflected well on their performance. The membrane with the highest porosity (0.1% *w*/*v* of Ti_3_ALC_2_, M1) achieved the highest pure water and protein solution fluxes of 182.5 and 148.7. The most hydrophilic membrane (M7) recorded the highest protein rejection and flux recovery ratio of 90.6, which was much higher than that of the pristine membrane, 26.2. MAX phase Ti_3_ALC_2_ is a potential material for antifouling membrane modification because of its protein permeability, improved water permeability, and outstanding antifouling characteristics.

## 1. Introduction

UF (ultrafiltration) technologies may effectively remove pathogens and turbidity at low pressures. Compact modular design, high water production, and excellent response to changes in water quality are all benefits of UF [1,2,3,4]. These benefits have led to the widespread use of UF membrane systems in many water treatment implementations. Polyvinylidene (PVDF) ultrafiltration membranes have received considerable attention in materials science due to their favorable properties. These properties include film formation, antioxidant activity, excellent aging resistance [5,6], high thermal and mechanical stability, and good chemical resistance [7]. The PVDF’s hydrophobic behavior, which has gained more consideration [8,9], may be addressed using membrane modification techniques such as surface modification, physical doping, or chemical grafting. Due to its simplicity, affordability, and ability to preserve the membrane’s intrinsic structure, combining nanomaterials with PVDF has become a common modification approach [10,11]. A unique hybrid PVDF-polyaniline (PANI) membrane incorporating graphene oxide (GO), for instance, was synthesized by Hifza et al. [8]. The manufactured membrane’s antifouling characteristics and solvent content were greatly enhanced by adding PANI-GO as a nanofiller. Similarly, Deng et al. [12] developed a modified blending method that incorporates the water-induced deposition of nanomaterials into PVDF phase reflection. The characterizations showed that the nanocomposite membranes were highly hydrophilic.

Recently, the field of membrane separation has garnered considerable attention for the novel family of 2D materials called “MAX phase Ti_3_ALC_2_” [13,14,15]. In order to produce MAX phase Ti_3_ALC_2_s, a form of 2D material, a layer is removed from their predecessor MAX phases. In this context, “M” refers to an early transition metal, “A” refers to a group element (often from the IVA or IIIA group), and “X” refers to carbon or nitrogen. Mn+1XnTx defines mxene Ti3C2Tx, and T stands for the functional groups (e.g., F, OH, or O). These have strong hydrophilic surfaces, electrical conductivity, a robust structure, and excellent chemical stabilities [16,17,18,19]. Separation membranes made of Ti3C2Tx have been widely applied in various applications, including gas separation, desalination, wastewater treatment, and ion sieving [20,21,22,23].

Subsequently, significant attention has been given to creating ultrafiltration membranes by incorporating carbon-based nanoparticles into polymers. These modified polymer membranes outperform standard polymer membranes regarding hydrophilicity, mechanical strength, and antifouling properties [24,25,26,27]. Han et al. [15] synthesized a novel 2D Ti3C2Tx mxene /PES composite membrane, which was shown to have superior hydrophilicity, increased water flux, and strong Congo red dye rejections. However, there are few investigations or extensive work on the MAX phase Ti_3_ALC_2_ membrane’s antifouling capabilities. In order to create a novel nanocomposite membrane with enhanced hydrophilicity and antifouling properties, MAX-phase Ti_3_ALC_2_ was inserted into the PVDF membrane in this study. The MAX-phase Ti_3_ALC_2_ nanoparticles were incorporated into the PVDF membrane using the phase inversion method. The impact of various MAX phase Ti_3_ALC_2_ concentrations on porosity, pore structure, and hydrophilicity was thoroughly assessed. The composite membranes’ water permeation, separation efficiency, morphology, and antifouling characteristics were studied.

## 2. Materials and Methods

### 2.1. Materials

Polyvinylidene fluoride (PVDF, 99.5%, 200,000 Mw, Jiangsu Frechem Co., Ltd., Nanjing, China) was used for preparing the membrane matrix. Polyvinylpyrrolidone (PVP, k30) was purchased from Sinopharm Co., Ltd., China. NMP (N-methyl-2-pyrroidone, 99.9%) was provided by Hebei Shengyuan Jinlong lmp. & EXP Co., Ltd., China. MAX phase (titanium aluminum carbide Ti_3_ALC_2_ nanoparticles, 99.95%) was obtained from Nanjing Aocheng Chemical Co., Ltd., Nanjing, China.

### 2.2. Membranes Preparation

**Figure 1:**
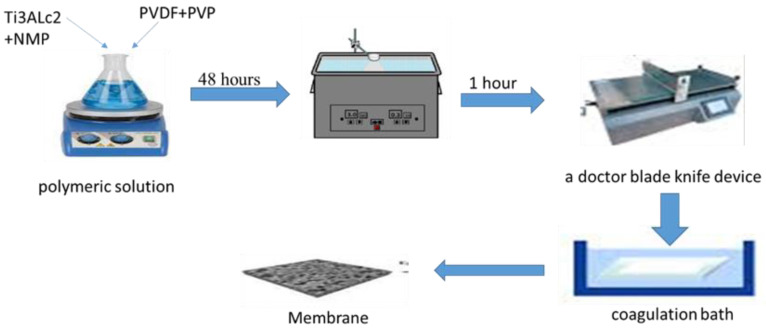
Preparation process of PVDF MMMs.

A polymeric solution was prepared by adding 20% of PVDF powder and 2% of PVP powder to N-Methyl-2-pyrrolidone (NMP) with a ratio of 78%. Then, the mixture was stirred for 24 h. After that, MAX phase Ti_3_ALC_2_ was added in different proportions (see Table 1) and stirred again for 24 h. After that, the solution was sonicated for 1 h. The solution was doped on a glass plate and cast using a doctor blade knife. The membrane was submerged in a coagulation bath of distilled water. The membrane was preserved in distilled water by replacing the water every two days. Later, these membranes were treated by immersing them in propanol alcohol with a purity of 99% and a concentration of 100% for two days. Finally, the membranes were dried with air and stored for later examination, as illustrated in Figure 1.

### 2.3. Characterization

#### 2.3.1. Characterization of Ti_3_C_2_Tx Nanoparticles

MAX PHASE Ti_3_ALC_2_ nanoparticles were qualitatively characterized by an X-ray diffractometer (Bruker D8 ADVANCE, Germany). The X-ray diffractometer was equipped with a monochromatic source of Cu Kα radiation (λ = 0.154 nm) operating from 10 to 90 at 40 mA and 40 °C. The morphology of MAX phase Ti_3_ALC_2_ nanoparticles was examined using field emission scanning electron microscopy (FE-SEM) (FEI, inspect F50, Japan).

#### 2.3.2. Characterization of PVDF/MAX Phase Ti_3_ALC_2_ Membranes

The morphology of produced membranes was studied using the Field TESCAN VEGA3 SB FE-SEM instrument (EO, Elektronen-Optik Services GmbH, Dortmund, Germany). Cross-sections of the membranes were prepared to utilize liquid nitrogen and coated with a thin platinum layer prior to being fixed on the specimen stubs using carbon tape. EDS (energy dispersive spectroscopy, Hitachi, type S4200N, Tokyo, Japan) was utilized to obtain an elemental mapping of the membrane surface. An appropriate silicon tip and an atomic force microscope (AFM) (Multimode 8, Bruker, Germany) were employed to capture the surface topographical profile of the membranes in contact mode. The cantilever flexes due to the rise and fall of the sample topography, and the amount of this deflection can be reflected by the photodetector as up–down signals. Measurements included an assessment of the vertical variation of the membrane’s surface topography (peak–valley), the lateral force (friction forces between tip and sample, which cause the cantilever torsion and can be reflected by the photodetector’s left–right signal), and the deflection. A statistical pore size distribution was established for the outer surfaces of each membrane using the IMAGER 4.31 program. The structural modification of the membrane caused by the injection of nanoparticles was examined using FTIR (Nicolet 6700, Fourier transform infrared spectroscopy/Thermo Electrons Corp., Madison, WI, USA).

The membranes’ hydrophilicity was assessed by measuring their water contact angles. Using the sessile drop technique, the membrane contact angle was determined. This was accomplished using the CAM110 optical contact angle device (Tainan, Taiwan). Every sample was evaluated in a minimum of five various places, and an average value was considered. To find out the porosity of the membranes, the membrane samples were cut into pieces of 2 cm^2^ and immersed in distilled water for 15 h. Wet films were retrieved from the water. Blotting paper was utilized to collect and weigh the extra droplets from the membrane surface. The membrane was oven dried for 12 h, and samples were dry-weighed. Using Equation (1) [28], the porosity of the membrane was calculated. For every membrane sample, five porosity estimations were collected, and the computations were averaged.

(1) $$\varepsilon \%=\frac{Ww-Wd}{A\times L\times \rho }$$

In which:

*Wd* and *Ww* = the membrane’s dry and wet weight, respectively;

*A* = membrane area (cm^2^);

ρ = water density = 1 g/cm^3^; and

*L* = thickness of the membrane (cm).

Equation (2) was used to calculate the mean pore size (*rm*) based on the porosity of the membrane and the flow of pure water:

(2) $$rm=\sqrt{\frac{\left(2.9-1.75\varepsilon \right)\times \left(8\mu LQ\right)}{A\times \varepsilon \times \Delta P}}$$

In this equation:

*Q* = collected volume of the pure water flux per unit time (m^3^/s),

μ = water viscosity,

*ε* = membrane porosity,

*DP* = operation pressure, and

*A* and *L* are the membrane’s effective area and thickness [29].

### 2.4. Membrane Performance

Laboratory-scale membranes with a membrane area of 13.69 cm^2^ were tested for their water permeability and separation performance in a homemade dead-end filtration apparatus. In order to maintain a steady flux, each membrane was compressed with 0.2 MPa of pressure and 30 min of pure water. The water flux was then tested when the pressure was lowered to its operating pressure of 0.1 MPa. Equation (3) was used to determine the permeate flux (*J*) [30]:

(3) $$J=\frac{V}{t\;\times \;A}\;$$

where:

*A* = efficient membranes area in the filtration cell (m^2^), and

*V* = collected permeate volume (m^3^) at time *t*.

After that, a bovine serum albumin (BSA) solution of a 1000 mg/L concentration was utilized to evaluate the membrane rejection (*R*) using Equation (4) [27]:

(4) $$R\left(\%\right)=\frac{Cf-Cp}{Cf}\times 100\%$$

where *Cp* and *Cf* are the BSA concentration in the permeate and feed solutions, respectively.

### 2.5. Antifouling Assessment

The solution depletion approach was used to assess the static protein adsorption onto membranes. The BSA was utilized as the model foulant. Membranes with a surface area of 13.69 cm^2^ were submerged in 1 g/L BSA of phosphate-buffered saline (PBS, 24 h, 25°C, 7.4 pH). Using a calibrated concentration curve, an ultraviolet spectrophotometer set to 280 nm was used to detect the concentration change before and after adsorption. The discrepancy is a representation of every membrane’s adsorption capacity. Following the water permeability and separation efficiency testing, the membrane was washed for 15 min in pure water to remove the protein that had been fouled. The water flux was then again measured in *Jw* (L/m^2^h). After that, the FRR (flux recovery ratios) were determined [31]:

(5) $$FRR\%=\frac{Jw,2}{Jw,1}\times 100\%$$

where 1 and 2 denote the flux before and after cleaning.

## 3. Results and Discussion

### 3.1. Characterization of the MAX Phase Ti_3_ALC_2_

**Figure 2:**
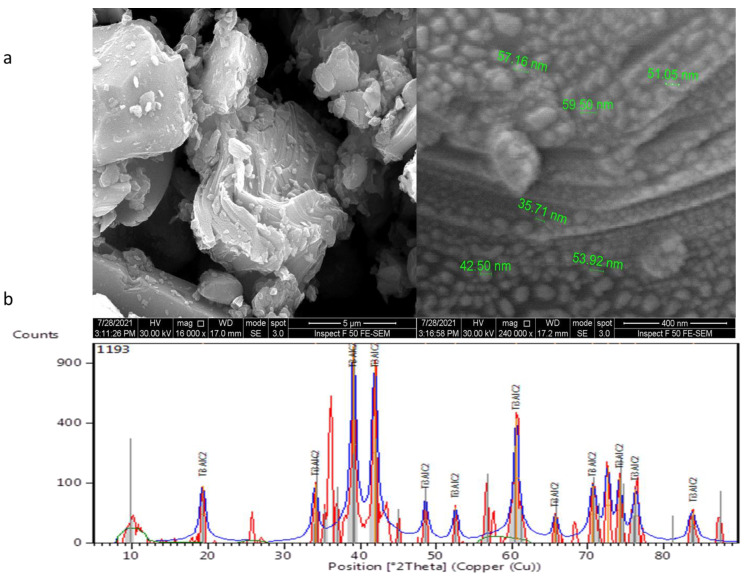
MAX phase Ti_3_ALC_2_ nanoparticles: (**a**) FESEM images and (**b**) XRD spectra.

X-ray diffraction examination showed pure MAX PHASE Ti_3_ALC_2_ NPs with diffraction peaks at 19.2818°, 34.0882°, 39.1207°, 41.9004°, 48.6569°, 52.6103°, 60.6025°, 65.7535°, 70.7231°, 72.6176°, 74.1642°, 76.2845°, and 83.7989°, as depicted in Figure 2a. These peaks are typical structural peaks of Ti_3_ALC_2_. Figure 2b shows FESEM images of MAX phase Ti_3_ALC_2_ nanoparticles. It was found that MAX phase Ti_3_ALC_2_ displays a classical 2D structure.

### 3.2. Characterization of PVDF/MAX PHASE Ti_3_ALC_2_ Membranes

**Figure 3:**
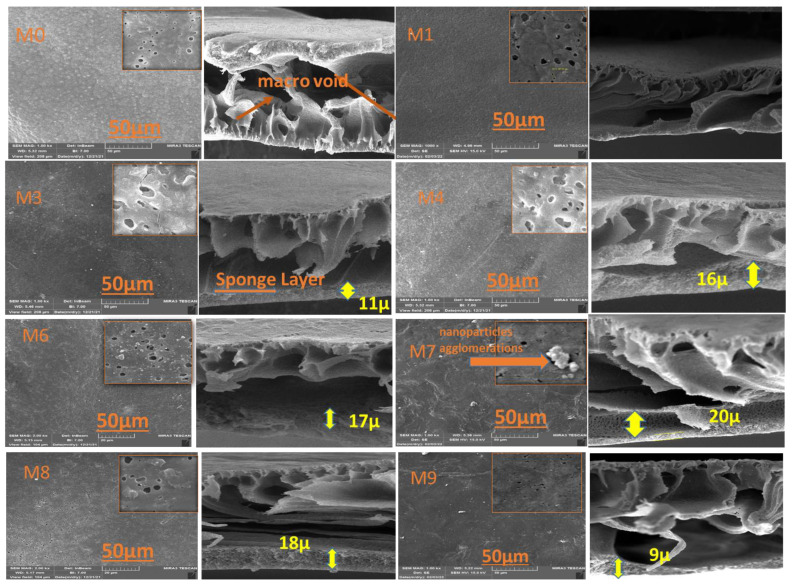
SEM images of the fabricated six PVDF/Ti_3_ALC_2_ membranes: M0, M1, M3, M4, M6, M7, M8, and M9.

The top surface and cross-section FESEM images of modified and unmodified membranes are presented in Figure 3. A thick asymmetric layer supported by multiple macro voids was found in all membranes, which showed a characteristic asymmetric skin microstructure (sub-layer). It could be noticed that the density and pore size at the top surface was improved by increasing the MAX PHASE Ti_3_ALC_2_’ load in comparison to the top surface of the neat PVDF membrane. Regarding the cross-section of FESEM images, it can be seen that two layers were obtained: one macro-void structure located near the top surface and a very thin sponge layer observed near the bottom surface. This is mainly due to the instantaneous solvent/non-solvent exchange rate throughout the fabrication of the membranes [32]. With the addition of different amounts of MAX phase Ti_3_ALC_2,_ the thickness of the thin sponge layer was increased. This phenomenon is attributed to the reduction in the exchange rate of solvent/non-solvent because of the effect of the embedded MAX phase Ti_3_ALC_2_ in the PVDF casting solution on the viscosity of polymer-NPs solution [33]. Increasing the MAX PHASE Ti_3_ALC_2_ increased the casting solution’s viscosity, which delayed the diffusion of organic solvent and water during the membrane fabrication. As a result, the sponge layer in the modified membrane increased, as shown clearly in Figure 3 (membrane M8). A similar phenomenon was reported in previous research [34]. It can be concluded that the incorporation of MAX PHASE Ti_3_ALC_2_ into the PVDF membranes altered the structure of the membrane. The change in membrane porosity, as visualized by SEM images, might seem to be subtle given the qualitative nature of this technique.

**Figure 4:**
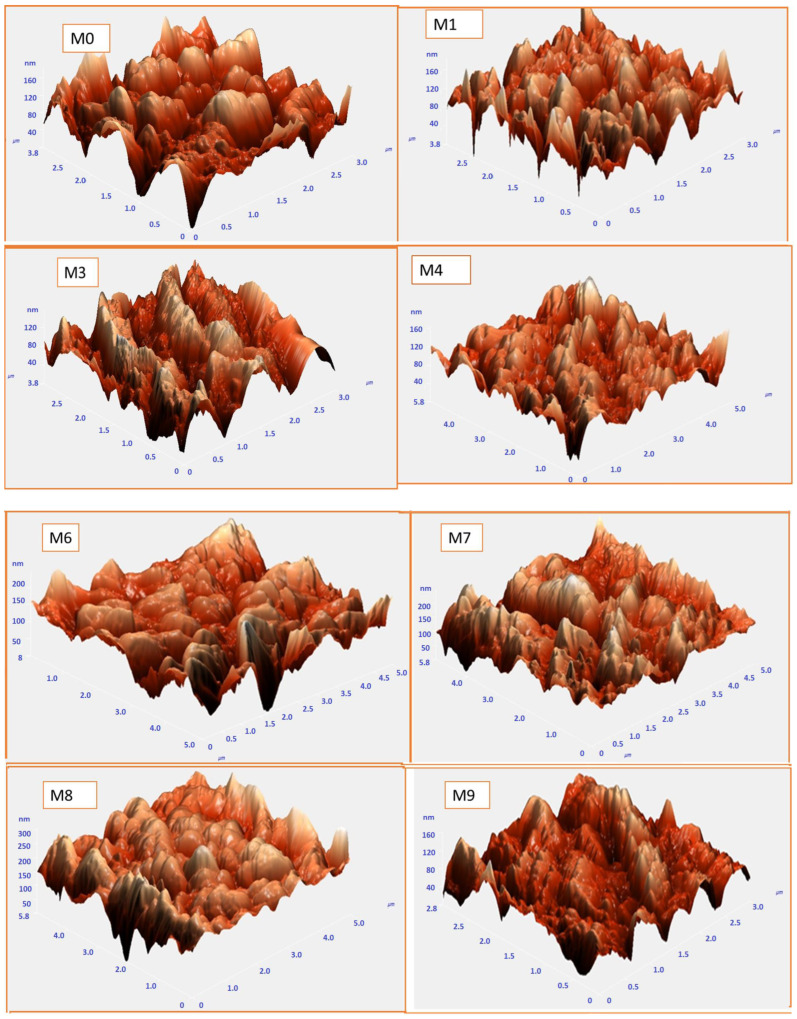
AFM images and the corresponding surface roughness for the PVDF/Ti_3_ALC_2_ membranes.

The brightest area in Figure 4 AFM images of the constructed membranes denotes the strongest point of the membrane surface. The valleys of membrane pores are also seen in the dark region. The average roughness (Sa), root-mean-square of the Z data (Sq), and the height variation between the tallest peak and the lowest valley (Sy) are some of the surface roughness metrics shown in Table 2. The surface roughness parameters of the composite membranes were decreased by the MAX phase Ti_3_ALC_2_ compared to the plain membrane. The nanofillers may have been consistently collocated in the membrane, which is one explanation. A smooth surface was created as a consequence of the many small peaks that occurred on the membrane surface rather than a few big ones [35,36]. To prevent foulants from penetrating the membrane pores, surface roughness must be reduced [37,38]. The PVDF- MAX PHASE Ti_3_ALC_2_ NPs’ membranes may exhibit improved antifouling capabilities as a consequence of all of these factors.

**Figure 5:**
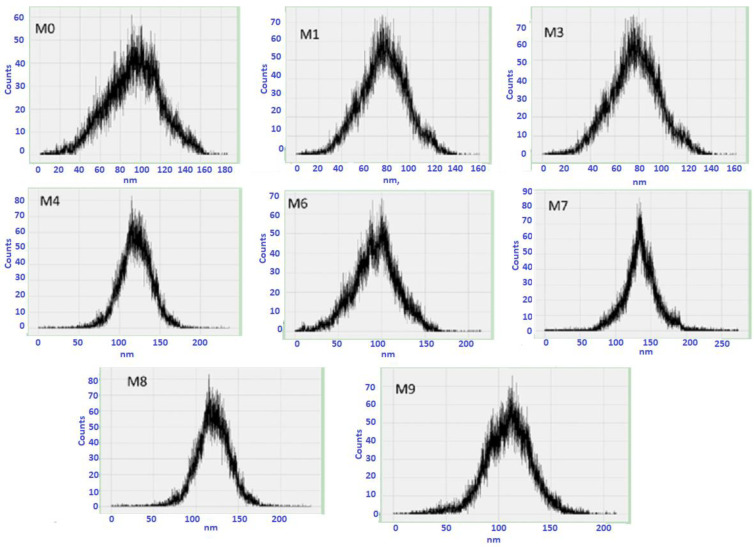
Pore size distribution of the modified and unmodified membranes.

**Figure 6:**
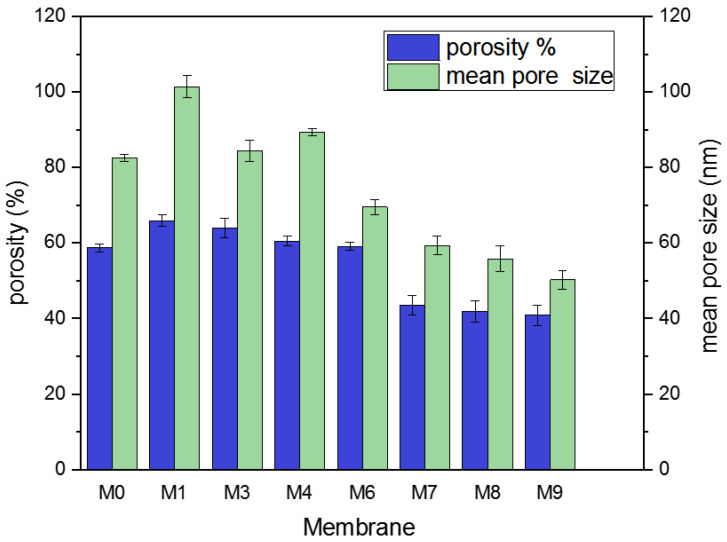
Mean pore size and porosity of different PVDF/Ti_3_ALC_2_ membranes.

The mean pore size (m) and distribution range of all membranes have also been determined using AFM (see Figure 5). The neat PVDF membrane has an 82.56 nm average pore size and a pore size distribution between 20 and 160 nm, whereas M1 has a mean pore size of 101.42 nm and a pore size distribution range of 20–130 nm. The addition of Ti_3_ALC_2_ at a low concentration (i.e., M1) increased the mean pore size and narrowed the distribution range. A further increase of Ti_3_ALC_2_ NPs led to a reduction in pore size while the pore distribution range continued to narrow. For example, the mean pore size of M3 was 84.47 nm, which was higher than M7 (59.31 nm). The increased quantity of nano-additives may improve casting solution viscosity, postpone the mixing–demixing process between the non-solvent and solvent, and bestow these reduced surface pore size palpable properties. These results agree with SEM results and experimentally measured porosity and mean pore diameters (Figure 6). All observations showed that adding Ti_3_ALC_2_ nanoparticles up to 0.3% resulted in increasing the membranes’ porosity, and further increases led to a lower porosity when the Ti_3_ALC_2_ content increased. The reduction of porosity and the mean pore size of the membrane could be attributed to the increase in polymer viscosity or membrane pore blockage caused by aggregation of the Ti_3_ALC_2_ nanoparticles [39].

**Figure 7:**
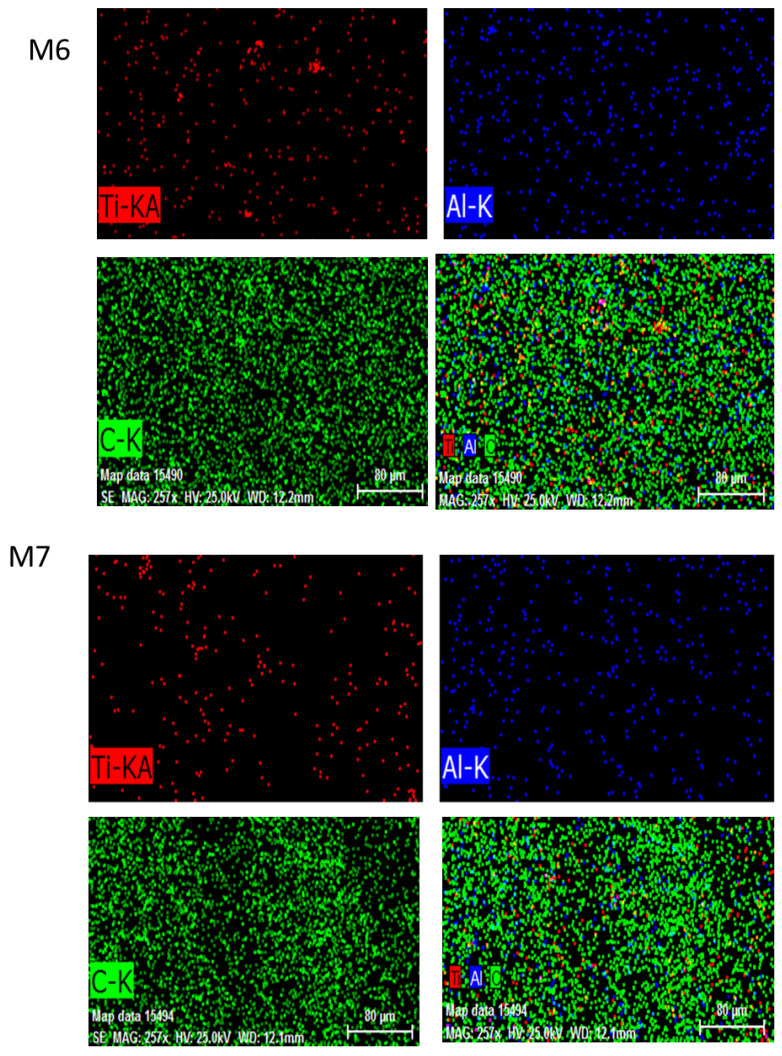

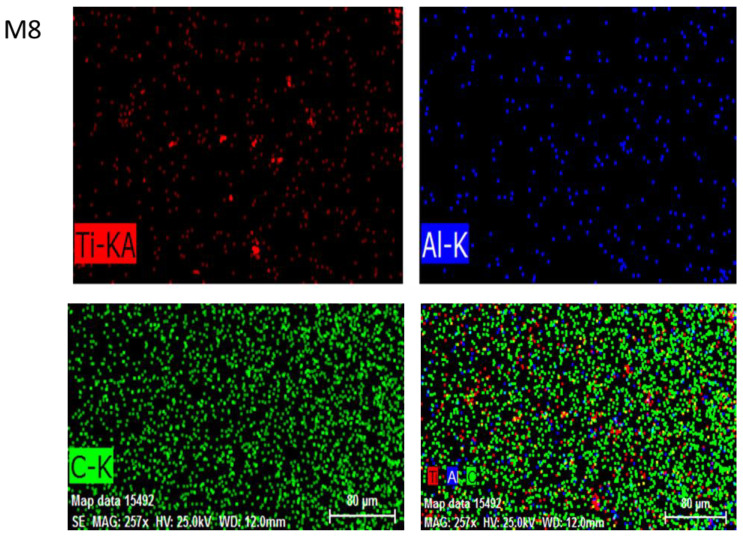
The energy dispersive spectroscopy (EDS) images of MAX phase Ti_3_ALC_2_-incorporated membranes M6, M7, and M8.

EDX was performed to analyze and confirm the modified membranes’ elemental compositions. C was primarily derived from PVDF and MAX phase Ti_3_ALC_2_ and Ti, whereas Al was acquired from MAX phase Ti_3_ALC_2_. The contents of the spectra and elements are given in Figure 7. The presence of Ti attested to the efficient incorporation of MAX phase Ti_3_ALC_2_ into the membrane [39].

**Figure 8:**
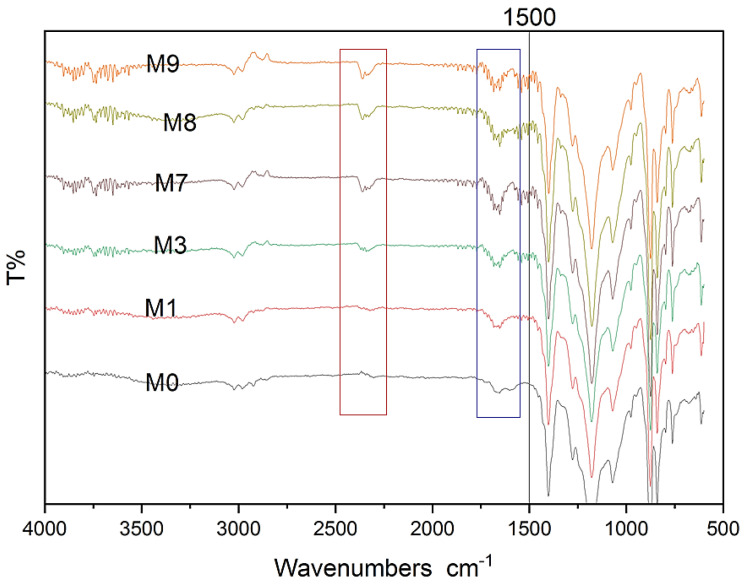
FTIR spectra for modified and unmodified membranes.

The chemical behavior of the PVDF membrane was analyzed using FTIR both before and after the addition of MAX phase Ti_3_ALC_2_ nanoparticles. The results are presented in Figure 8. There is a clear increase in peak intensity at 1650, 2349, and 3767 cm^−1^ after adding Ti_3_ALC_2_. These peaks correspond to –C=O, –CH, and –OH groups. Recent studies also detected these peaks that synthesized pristine and composite MAX phase membranes [40,41,42]. The membrane’s spectra showed that the introduction of MAX phase Ti_3_ALC_2_ nanoparticles did not alter the features of the vibrational bands, suggesting that the membrane’s chemical composition and non-covalent interaction with MAX phase Ti_3_ALC_2_ remained unaltered.

**Figure 9:**
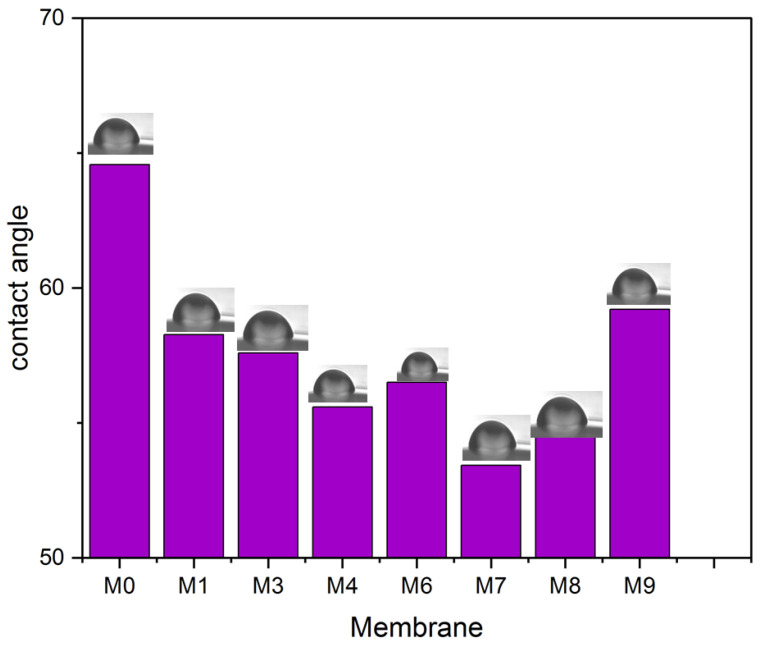
Water contact angle for all PVDF/MAX PHASE Ti_3_ALC_2_ membranes.

Hydrophilicity is among the essential criteria for a membrane utilized for filtration and is directly associated with the membrane’s antifouling capabilities. Figure 9 illustrates the measurement of the water contact angle for all membranes. The neat PVDF membrane exhibited the greatest water contact angle of all the membranes. With the increase of MAX phase Ti_3_ALC_2_ content, the water contact angle lowered to 53.43°, which indicates the improvement in the hydrophilicity of the membranes [42]. Increasing the percentage of Ti_3_ALC_2_ beyond 0.7% results in increasing the water contact angle of the membranes. This could not be explained by surface roughness, as it decreased after increasing the number of nanoparticles added. It was noticed that the intensity of the FTIR peaks for the oxygen-containing functional groups (e.g., C=O) that normally correlate well with hydrophilicity decreased after the increasing Ti_3_ALC_2_ beyond 0.7%. When the Ti_3_ALC_2_ nanoparticles increased in the PVDF casting solution, they could agglomerate on the surface of the membrane, which, in turn, may overshadow the effect of the functional groups and their effects on membrane water affinity.

**Figure 10:**
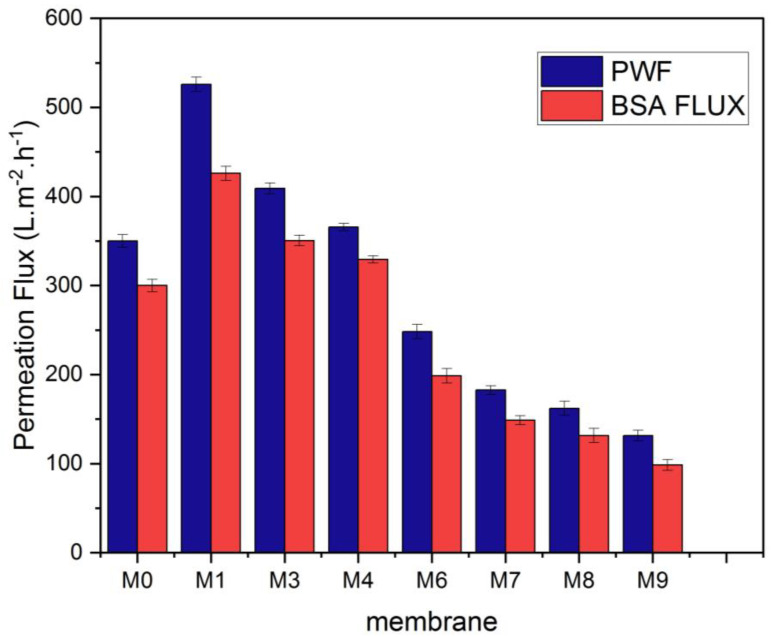
Pure water and BSA (bovine serum albumin) flux of the MAX phase Ti_3_ALC_2_/(PVDF) membranes.

**Figure 11:**
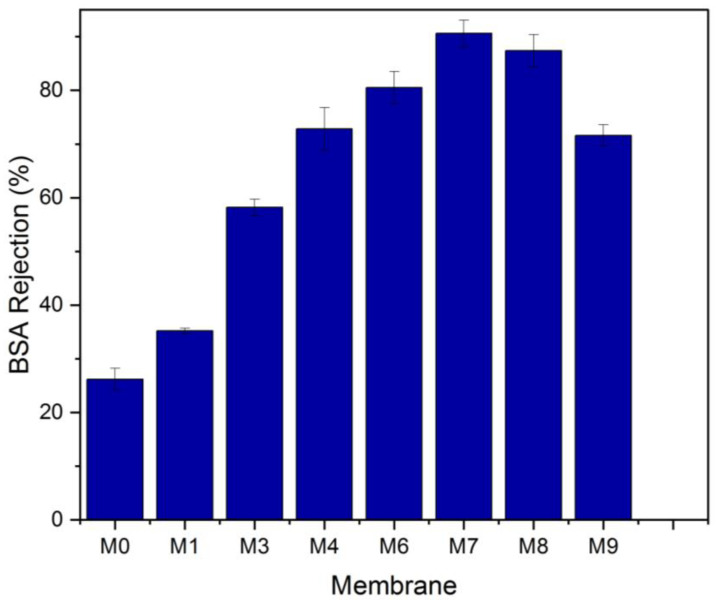
Bovine serum albumin (BSA) rejection of the neat PVDF and MAX phase Ti_3_ALC_2_/PVDF membranes.

At a pressure of 0.1 MPa, the pure water flux (PWF) was estimated. Figure 10 illustrates how the content of MAX phase Ti_3_ALC_2_ affects the performance of PVDF/MAX phase Ti_3_ALC_2_ membranes. The pure water flux of the PVDF/MAX composite Ti_3_ALC_2_ membrane rose with the addition of MAX phase Ti_3_ALC_2_ up to 0.4 *w*/*w*%; however, further increases in the concentration of the additives beyond this percentage led to an increase in the contact angle and a decrease in porosity. This change reflects well on the pure water flux of the membrane. The increase of MAX phase Ti_3_ALC_2_ beyond 0.4 *w*/*w*% may induce pore clogging of the membrane, leading to a decrease in the pure water flux [43]. As usually happens with hydrophilic additives, as reported in the literature [19,23,27], their molecules could be occluded or entrapped between the polymer chains. The same pattern was observed using a BSA solution with a small decline in the permeation flux, as shown in Figure 9. Solutes are deposited on the membrane surface and pores, causing membrane contamination and decreased flow throughout filtration [44,45]. Although membrane fouling is unavoidable, it may be mitigated to some extent by using the right solutions, such as inserting a hydrophilic substance into the membrane. Adding MAX phase Ti_3_ALC_2_ in this study enhanced the membrane’s hydrophilicity and contamination. Using bovine serum albumin (BSA) as a representative fouling agent, the antifouling capabilities of the membranes were assessed. Before and after the filtration of the BSA solution, the relative pure water flux (PWF) was computed and given as the solution recovery ratio. Figure 11 shows that the BSA rejection of neat PVDF membrane was low at about 28%, due to its hydrophobic nature, whereas with the addition of MAX PHASE Ti_3_ALC_2,_ the rejection of BSA increased to a maximum of 90% [46,47].

### 3.3. Antifouling Performance

The efficiency of separation and long-term operation are both greatly impacted by membrane fouling. Membrane fouling forms detrimental to membrane efficiency include pore blockage, concentration polarization, and cake layer formation [48]. The membrane surface hydrophobicity illustrates the PVDF membranes’ poor antifouling efficacy, despite the reasons for fouling membranes being quite complicated. An efficient and practical way to improve the antifouling capabilities of the membrane was to introduce hydrophilic MAX phase Ti_3_ALC_2_ into the membrane [49,50,51,52,53,54]. With the increasing content of MAX phase Ti_3_ALC_2_, the amount of BSA adsorbed on the surface of the composite membrane decreased with a minimum value of M6 (275 µg·cm^−1^). The amount of protein adsorbed reduced as affinity rose because the membrane surface shrank and there was less contact between the protein and the membrane [55,56,57,58,59,60,61].

**Figure 12:**
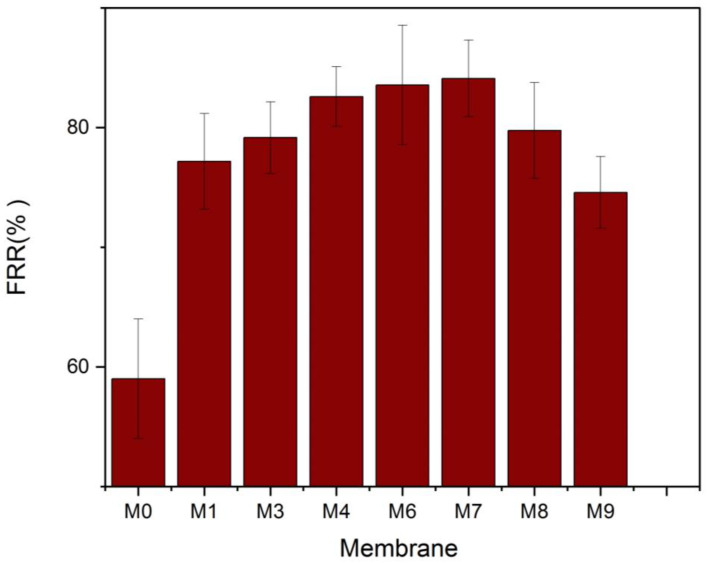
Water flux recovery ratio of the MAX phase Ti_3_ALC_2_/PVDF membranes.

The FRR% was determined to assess the antifouling capabilities of the membranes further, and the findings are illustrated in Figure 12. The FRR for the plain PVDF membrane was 59% lower than that of the other nanocomposite membranes. Due mainly to its increased hydrophilicity, the Membrane M7 had the greatest flux recovery ratio (84.1%). The interaction between the foulants and the membrane surface determines whether fouling is irreversible or reversible. Reversible fouling is induced by weak interactions between the membrane surface and the fouling particles, which are easily removed. It appears that most of the accumulated BSA on M7 was reversible fouling. The foulants’ attachment to the UF membrane is dictated by its rejection mechanism (molecular sieving and adsorption) and the nature of the interaction of foulants with the membrane surface (weak, physical interaction or strong, chemical bonding). Since protein adsorption onto surfaces is governed mainly by non-specific interactions such as hydrophobic–hydrophobic interaction [61], it makes sense that BSA adhesion onto the UF membrane with the highest hydrophilicity (lowest contact angle) would be the weakest. Consequently, this led to the best FRR.

**Figure 13:**
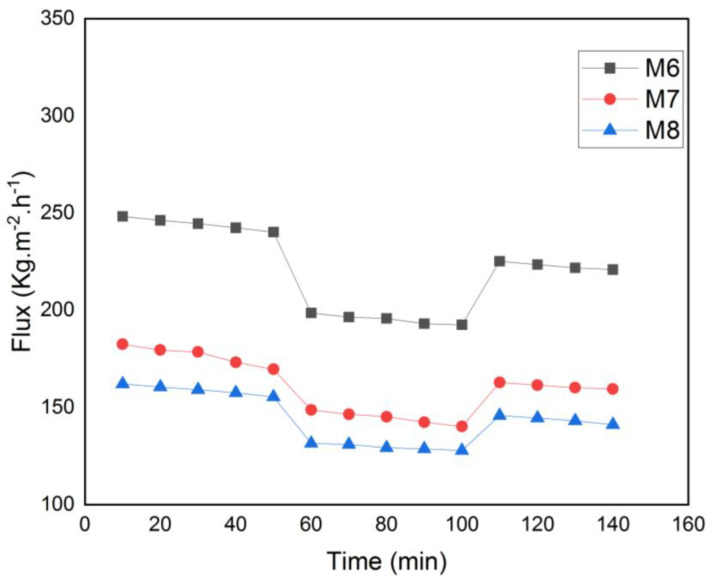
Time-dependent fluctuation of BSA solution flux for M6, M7 and M8.

To assess the impact of MAX phase nanoparticles on membrane antifouling characteristics and reusability, a time-dependent flow test of the membranes was conducted. The pure water flow before and after protein filtration is shown in Figure 13. Pre-compaction of the membranes caused a modest reduction in pure water flow for all the membranes during the initial filtering stage. When the BSA solution was utilized as feed, the water flow then decreased. The membrane’s pores were then partially blocked by the BSA protein, which decreased the amount of water that could pass through the membrane. After that, deionized water was used to wash each membrane. After physical cleaning, the water flow was once again monitored to see how well the restoration process worked.

Table 3 shows how the results of this study were compared to those of other PVDF ultrafiltration studies that used the addition of different nanoparticles to improve antifouling properties. Due to the increased hydrophilicity brought about by the MAX membrane phase Ti_3_ALC_2_, the MAX membrane Ti_3_ALC_2_ demonstrated excellent protein rejection and water permeation efficiency.

## 4. Conclusions

This study used composite MAX phase Ti_3_ALC_2_ to synthesize fouling-resistant PVDF membranes for UF application. Different types of Ti_3_ALC_2_ were applied to identify the nanoparticle/polymer ratio. Various advanced surface and structure chemistry analyses were employed to study and carefully track the changes brought about by the additives. The performance of the nanocomposite membranes was evaluated using pure flux and BSA reject tests. The addition of Ti_3_ALC_2_ increased membrane roughness, hydrophilicity, and mean pore diameter, reducing the pore size distribution spectrum. However, these effects occurred up to a percentage of 0.3 *w*/*v*. After, there were irregular reverse trends in these properties. This was reflected in the membrane performance, where pure water and BSA solution fluxed. As for BSA rejection and FRR, M7 recorded the best results, likely due to its high hydrophilicity compared to other membranes. The findings of this study show promising results of using Ti_3_ALC_2_ as a potential additive for reducing PVDF fouling, which merits further investigation into the performance of the produced mixed matrix membrane with complex matrix samples for long-term tests.

## Figures and Tables

**Table 1:** Composition of the casting solution of the modified and unmodified membranes.

Sample Code	Composition			
	PVDF (wt.%)	PVP (wt.%)	NMP (wt.%)	MAX PHASE Ti_3_ALC_2_ (wt.%)
M0	20	2	78	0
M1	20	2	77.9	0.1
M3	20	2	77.7	0.3
M4	20	2	77.6	0.4
M6	20	2	77.4	0.6
M7	20	2	77.3	0.7
M8	20	2	77.2	0.8
M9	20	2	77.1	0.9

**Table 2:** Parameters of the membranes’ surface roughness.

No. Sample	Ten-Point Height, Sz (nm)	Average Roughness, Sa (nm)	Root Mean Square, Sq (nm)
M0	92.8313	21.86	27.3507
M1	106.626	20.3984	26.0354
M3	79.0782	16.2117	20.5072
M4	114.913	15.3444	19.8073
M6	114.913	15.3444	19.8073
M7	61.9802	14.8291	18.7414
M8	80.0782	14.2117	18.5072
M9	76.913	13.3444	16.8073

**Table 3:** Comparison of different antifouling PVDF-based membranes.

Membrane	Optimum Dosage (wt.%)	Contact Angle (°)	Foulant	Water Flux (L m^−2^ h^−1^)	Rejection (%)	Ref.
PVDF-ZnO	1.5	63.2	RW	147.2	93	[35]
PVDF-TiO_2_	4	60.7	BSA	103.5	85.6	[51]
PVDF-OMWCNTs	1	66.8	BSA	119	86.9	[53]
PVDF-GO	1	66.4	BSA	163	83.7	[55]
PVDF-SiO_2_	3	56.7	BSA	198	94.5	[56]
PVDF-Fe_3_O_4_	25	_	BSA	65.6	93	[57]
PVDF-GO/OMWCNTs	1	48.6	BSA	203	81.6	[58]
PVDF-GO/TiO_2_	1	61	BSA	487.8	92.5	[59]
PVDF-rGO/TiO_2_	1.05	69	BSA	221	99	[60]
PVDF—MAX PHASE Ti_3_ALC_2_	0.6	56.71	BSA	248.3	80.5	Current work
PVDF—MAX PHASE Ti_3_ALC_2_	0.7	53.43	BSA	182.5	90.6	
PVDF—MAX PHASE Ti_3_ALC_2_	0.8	56.5	BSA	162.1	87.4	

## Data Availability

Not applicable.
